# Editorial: Inhibitors of CDK family: New perspective and rationale for drug combination in preclinical models of solid tumors

**DOI:** 10.3389/fonc.2023.1180650

**Published:** 2023-03-28

**Authors:** Andrea Cavazzoni, Arianna Palladini

**Affiliations:** ^1^ Department of Medicine and Surgery, University of Parma, Parma, Italy; ^2^ Department of Molecular Medicine, University of Pavia, Pavia, Italy

**Keywords:** CDK, solid tumors, pre-clinical, combination, Rb

The CDK family includes enzymes involved in cell cycle progression and transcriptional regulation. CDK catalytic activity is strictly dependent on cyclin binding. Although various CDKs and cyclins have been identified, specific CDK-cyclin complexes are mandatory to control cell cycle progression, i.e., through the G_1_, S, G_2_, and M phases. Thus, CDK family activity can be modulated by cyclin partners and cyclin-dependent kinase inhibitors (CKIs).

The activity of CDK4/6 is mainly addressed to the tumor suppressor protein retinoblastoma (Rb) that causes the inactivation of Rb activity through hyperphosphorylation and the subsequent progression of the cell cycle from the G_1_ to the S phase. In detail, the cyclin D-CDK4/6 kinase complex phosphorylates Rb, thus removing the control on the E2F transcription factor, allowing the transcription of genes critical for DNA replication and cell cycle progression from the G_1_ to the S-phase. On the contrary, in its non-phosphorylated state, Rb binds the E2F transcription factor, actively suppressing the G_1_-S progression.

A high level of Rb protein phosphorylation has been detected in several tumors and associated to the high expression of the cyclinD-CDK4/6 complexes. Thus, targeting these complexes is an attractive strategy to fight cancers. This strategy is currently approved for the treatment of estrogen receptor (ER)-positive, human epidermal growth factor receptor (Her) 2-negative breast cancer in combination with the aromatase inhibitor letrozole. Furthermore, several experimental data suggest the opportunity to extend the therapeutic strategy to other solid tumors. Moreover, encouraging results have shown that other members of the CDK family could be an attractive target to fight different tumors.

The Research Topic titled “*Inhibitors of CDK family: New Perspective and Rationale for Drug Combination in Preclinical Models of Solid Tumors*” focuses on the recent progress made with the aim of improving the efficacy of cyclin–CDK complex inhibitors. The Special Issue published nine articles, consisting of seven original articles and two review articles.

The paper by Digiacomo et al. described the efficacy of combining the CDK4/6 inhibitors abemaciclib or palbociclib with the antiangiogenic factor lenvatinib in a panel of human HCC cell lines. The simultaneous combination presented a significant antiproliferative effect compared to single therapy and different schedules of treatment. Growth inhibition was associated with a marked c-myc downregulation and a shutdown of the main intracellular signaling pathways. Moreover, the planned combo therapy was responsible for the irreversible inhibition of tumor growth due to the acquisition of a senescent phenotype.

Chilà et al. analyzed the role of CDK12, a member of the CDK family, involved in the phosphorylation and activation of the RNA polymerase II, the most important enzyme regulating the transcriptional process in eukaryotic cells. CDK12 is mainly inactivated in ovarian cancer, but, to date, the precise contribution of CDK12 to tumor progression still needs to be clarified. By generating stable CDK12 KO ovarian cancer cells, the authors observed a significant shutdown of cell growth in both *in vitro* and *in vivo* models, suggesting an oncogenic driver role of CDK12. Moreover, DNA content and chromosome doubling was observed in the absence of CDK12 over parental cells, suggesting an involvement of CDK12 in the maintenance of genome integrity.

As the CDK family is composed of numerous protein kinases involved in cell cycle regulation, the discovery and evaluation of new agents targeting specific CDK family members is encouraged.

In this context, the paper proposed by (1) described the activity of a new compound against the CDK1 inhibitor. This molecule is an active metabolite of the antiparasitic drug nitazoxanide (NTZ) and exhibited antiproliferative properties, with G_2_/M cell cycle arrest and apoptosis induction, on a panel of glioma cells.

Sun et al. proposed an attractive manuscript proving that high CDK6 mRNA and protein levels predict poor prognosis in patients with advanced bladder carcinoma (BLCA). Moreover, they proposed that bladder cancer cells with high CDK6 protein levels display high sensitivity to CDK4/6 inhibitors such as palbociclib and ribociclib. Despite the interesting results, the authors suggested that it is still arbitrary to use CDK6 mRNA as markers for patient selection. They underlined the critical role of the tumor microenvironment and the need to develop 3D cultures from tumor patients for precise prediction of the sensitivity to CDK4/6 inhibitors.

Recently, the third-generation retinoid adapalene (ADA) has been emerging as a potent anticancer agent, and using ADA in combination with existing therapeutic regimens may improve its effectiveness and minimize toxicities and drug resistance.

Two research articles focused on the role of ADA in prostate and triple negative breast cancer (TNBC).

Nong et al. (2) observed that ADA suppressed the proliferation of prostate cancer cells both *in vitro* and *in vivo*, with a reduction of metastatic lesions at the bone marrow level.

Moreover, ADA triggered S-phase arrest in prostate cancer cells by inhibiting CDK2, Cyclin A2, and Cyclin E1 and promoted apoptotic cell death because of BAX upregulation.

The second research article assessed the anticancer efficacy of ADA as a combination strategy with the PI3K inhibitor (GDC-0941) in *in vitro* TNBC models. This combination exerted a synergistic effect in term of inhibition of cell proliferation, inducing apoptosis, suggesting that this therapeutic option could be valuable for patients with TNBC.

Interestingly, the authors observed that CDK2 is the main target of ADA. CDK2 is often overexpressed in breast tumors and plays a key role in controlling cell cycle regulation, in particular the S-phase, making CDK2 an attractive therapeutic target. Molecular docking studies revealed that ADA binds with CDK2, inducing a significant reduction of CDK2 protein levels, confirming that S-phase arrest is a direct consequence of CDK2 abrogation by ADA treatment (3).

Recently, different immunomodulatory effects of CDK4/6 inhibition have been proposed, such as the increase of immune cells in the tumor microenvironment, which strengthens the antitumor immune response; these studies enforce the modulatory role of CDK inhibitors toward immune cells.

As reported by (4), palbociclib resulted in reversibly inhibiting the cell growth of human CD3^+^ T cells, with a consequent decrease of the pro-inflammatory cytokines IFN-γ and TNF-α, suggesting a shut-down of T-cell killing activity. These results may have consequences for the planning of treatment based on the simultaneous combination of CDK4/6 inhibitors and T-cell-based cancer immunotherapeutic strategies, and they suggest a palbociclib-free period for an effective immunotherapy approach.

The employment of CDK inhibitors could be also expanded to sarcoma patients. As reported in the review by Higuchi et al., palbocilcib addiction significantly reduced PDX growth in patients with sarcomas from different origins. The regression of PDX is achieved by addiction to different drugs, such as the multikinase inhibitor sorafenib and the recombinant methioninase, suggesting that the combination with palbociclib could be a promising therapeutic option to improve sarcoma therapy in the clinic.

The study of cell cycle control in the sarcoma histotype will be critical for the progress of combination treatment of CDK inhibitors with targeted therapies or immunotherapeutic strategies. Emerging data have confirmed the CDKN2A gene as the most altered gene in bone sarcoma and soft tissue sarcoma patients, confirming the biological value of p16INK4a-CDK4/6-pRb signaling in sarcomas.

At present, few phase I and II clinical trials are focusing on the addiction to CDK inhibitors in sarcoma patients, and the majority address CDK drugs in monotherapy.

Only three phase II clinical trials were based on the combination of CDK compounds with other anticancer drugs (chemotherapy) or with immune checkpoint inhibitors, the last one being the most promising strategy, based on results from breast cancer patients treated with letrozole, palbociclib, and pembrolizumab (Merlini et al.).

## Author contributions

All authors listed have made a substantial, direct, and intellectual contribution to the work and approved it for publication.

## References

[1] Huang S Xiao J Wu J Liu J Feng X Yang C. Tizoxanide promotes apoptosis in glioblastoma by inhibiting Cdk1 Activity. Front Pharmacol (2022) 13:895573. doi: 10.3389/fphar.2022.895573 PMC9174573 35694267

[2] Nong HB Zhang YN Bai YG Zhang Q Liu MF Zhou Q. Adapalene inhibits prostate cancer cell proliferation *in vitro* and *in vivo* by inducing DNA damage, S-Phase cell cycle arrest, and apoptosis. Front Pharmacol (2022) 13:801624. doi: 10.3389/fphar.2022.801624 PMC8902295 35273495

[3] Mehraj U Wani NA Hamid A Alkhanani M Almilaibary A Mir MA. Adapalene inhibits the growth of triple-negative breast cancer cells by S-Phase arrest and potentiates the antitumor efficacy of Gdc-0941. Front Pharmacol (2022) 13:958443. doi: 10.3389/fphar.2022.958443 PMC9393306 36003501

[4] Arndt C Tunger A Wehner R Rothe R Kourtellari E Luttosch S. Palbociclib impairs the proliferative Capacity of activated T cells while retaining their cytotoxic efficacy. Front Pharmacol (2023) 14:970457. doi: 970457 10.3389/fphar.2023.970457 PMC9935825 36817127

